# Novel Nano-Sized MR Contrast Agent Mediates Strong Tumor Contrast Enhancement in an Oncogene-Driven Breast Cancer Model

**DOI:** 10.1371/journal.pone.0107762

**Published:** 2014-10-08

**Authors:** Per-Olof Eriksson, Emil Aaltonen, Rodrigo Petoral, Petter Lauritzson, Hideki Miyazaki, Kristian Pietras, Sven Månsson, Lennart Hansson, Peter Leander, Oskar Axelsson

**Affiliations:** 1 Spago Nanomedical, Lund, Sweden; 2 Department of Laboratory Medicine Lund, Division of Translational Cancer Research, Lund University, Lund, Sweden; 3 Department of Medical Radiation Physics, Lund University, Skåne University Hospital, Malmö, Sweden; 4 Department of Radiology, Lund University, Skåne University Hospital, Malmö, Sweden; 5 Department of Medical Biochemistry and Biophysics, Division of Vascular Biology, Karolinska Institutet, Stockholm, Sweden; University of Missouri-Columbia, United States of America

## Abstract

The current study was carried out to test the potential of a new nanomaterial (Spago Pix) as a macromolecular magnetic MR contrast agent for tumor detection and to verify the presence of nanomaterial in tumor tissue. Spago Pix, synthesized by Spago Nanomedical AB, is a nanomaterial with a globular shape, an average hydrodynamic diameter of 5 nm, and a relaxivity (r_1_) of approximately 30 (mM Mn)^−1^ s^−1^ (60 MHz). The material consists of an organophosphosilane hydrogel with strongly chelated manganese (II) ions and a covalently attached PEG surface layer. *In vivo* MRI of the MMTV-PyMT breast cancer model was performed on a 3 T clinical scanner. Tissues were thereafter analyzed for manganese and silicon content using inductively coupled plasma-atomic emission spectroscopy (ICP-AES). The presence of nanomaterial in tumor and muscle tissue was assessed using an anti-PEG monoclonal antibody. MR imaging of tumor-bearing mice (n = 7) showed a contrast enhancement factor of 1.8 (tumor versus muscle) at 30 minutes post-administration. Contrast was retained and further increased 2–4 hours after administration. ICP-AES and immunohistochemistry confirmed selective accumulation of nanomaterial in tumor tissue. A blood pharmacokinetics analysis showed that the concentration of Spago Pix gradually decreased over the first hour, which was in good agreement with the time frame in which the accumulation in tumor occurred. In summary, we demonstrate that Spago Pix selectively enhances MR tumor contrast in a clinically relevant animal model. Based on the generally higher vascular leakiness in malignant compared to benign tissue lesions, Spago Pix has the potential to significantly improve cancer diagnosis and characterization by MRI.

## Introduction

Magnetic resonance imaging (MRI) offers several advantages over other methods for soft tissue tumor visualization and quantification, primarily high spatial resolution, good soft tissue contrast and no involvement of ionizing radiation. MRI has therefore come to play a pivotal role in the diagnosis and monitoring of cancer. Contrast agents (CAs) improve both the specificity and sensitivity of MRI investigations. Today's CAs are based on small molecules containing gadolinium (Gd). These CAs extravasate very quickly into all tissues and therefore the imaging method often requires fast data acquisition, so-called dynamic imaging [1], [2]. The fast data acquisition comes at the cost of spatial resolution, which contributes to a significant number of false-positive findings [3]–[5]. According to a meta-analysis of MRI accuracy in breast cancer staging by Houssami et al., the positive predictive value was 66% (95% CI: 52–77%) [6].

The endothelial cells of capillaries of most tissues form a barrier which limits passage of macromolecules. Tumor vasculature however, is leaky to large molecules. Additionally, tumors frequently lack functional lymphatic vessels. Collectively, these features lead to an effect called enhanced permeability and retention (EPR) as first described by Maeda et al [7]. Exploiting the EPR-effect, nanoparticles designed with a suitable size will remain inside healthy vessels but selectively leak into tumor tissue, thereby enabling selective tumor contrast enhancement and extending the time window for analysis compared with what is possible with the small-molecule CAs used today.

Several attempts at producing macromolecular CAs for tumor imaging are described in the literature [8], but so far none has been successful in the clinic. The great majority of macromolecular CAs has been based on Gd, which is associated with a risk of nephrogenic systemic fibrosis (NSF), a serious side effect, in patients with renal insufficiency. Lately, there have also been concerns for retention of Gd in the body after repetitive use of Gd-containing CAs [9]. To circumvent the problem of NSF while still offering positive contrast, manganese (Mn), which is a trace element essential for normal body function and development, has been proposed as an alternative to Gd [10], [11].

The aim of the current study was to test the *in vivo* properties of Spago Pix, a new macromolecular Mn-based MR contrast agent. Performing MRI analysis of mice from a clinically relevant model (MMTV-PyMT) of breast cancer, we show that Spago Pix selectively leaks into tumor tissue and enables excellent tumor contrast enhancement. Immunohistochemistry and chemical analysis confirm localization of Spago Pix in tumor tissue but not in normal tissue.

## Material and Methods

### Animals

Murine mammary tumor virus-polyoma middle T (MMTV-PyMT) mice for the imaging studies were bred and housed at the Department of Medical Biochemistry and Biophysics, Division of Vascular Biology, Karolinska Institutet, Stockholm, Sweden. For pharmacokinetic analysis, Naval Medical Research Institute (NMRI) mice and Sprague Dawley (SD) rats (Charles River Laboratories, Germany) were housed at Adlego Biomedical AB (Solna, Sweden).

### Ethics statement

All animal experiments were carried out in accordance with the recommendations of the Swedish Animal Welfare Act. The protocol was approved by the Regional Ethical Committees for Animal Research at Stockholm and Lund, Sweden (permit ID; N96/11, N200/12 and N258/11). MRI analyses were performed under anesthesia, and efforts were made to minimize animal discomfort. Following MRI and pharmacokinetic analysis, animals were euthanized by cervical dislocation. All animals were housed under standard animal facility conditions with free access to standard rodent food and water.

### Nanomaterial

Synthesis of the Spago Pix nanomaterial [12] was initiated by dissolving 12.5 mmol 1,1-bis(triethoxysilylpropyl)-1,1- bis(dimethylphosphonato)methane in 250 ml aqueous 80% ethylene glycol. The mixture was heated for 26 h at 114°C (inner temperature) whereupon mPEG 6–9 propyltriethoxy silane was added (5 mmol) and heating at 114°C was continued for 5 h. The solution of the PEGylated nanostructure was adjusted to pH 4.5 with 1 M Trizma base and 1.1 g manganese (II) chloride tetrahydrate was added. Thereafter, the solution was heated at 100°C for 112 h. The resulting solution was subjected to ultrafiltration using Millipore Centriprep centrifugal filter units. First, the solution was passed through a 100 kDa NMWC filter to remove high molecular weight nanomaterial. Secondly, the nanomaterial was filtered on a 10 kDa NMWC filter followed by repeated washing with MilliQ water as to remove unpolymerized monomer and unbound manganese. The diafiltered material was then concentrated on a 10 kDa NMWC filter and analyzed for elemental composition using ICP-AES. Concentrated nanomaterial was prepared for injection by electrolytical balancing, adjustment of pH to 7.4, osmotic equilibration using mannitol and dilution to a manganese concentration of 2 mM. The nanomaterial was further characterized by gel permeation chromatography (GPC), dynamic light scattering (DLS), zeta potential and relaxivity measurements. The size distribution and average hydrodynamic diameter were measured by GPC by comparing with protein standards of known size (bovine serum albumin and myoglobin). Hydrodynamic diameter and zeta-potential were measured by DLS on a Malvern Zetasizer Nano ZS equipped with a back-scattering detector (173°) (Malvern Instruments Ltd, Malvern, England). For zeta potential measurements the parameters were 25°C and 150 V. Longitudinal relaxivity (r_1_) and transverse relaxivity (r_2_) were measured using a Minispec mq60 NMR analyzer (60 MHz, Bruker Corporation, Billerica, MA) at 37°C. Samples were diluted in either MilliQ water or human blood plasma (Sigma Aldrich, St. Louis, MO).

### Elemental analysis by ICP-AES

After imaging, pieces of tumor, usually from beneath the fore limbs, and skeletal muscle from the hind limbs were excised and frozen. Tumor and skeletal muscle from one non-treated animal was included as a control. Since Mn is the paramagnetic component providing the MR contrast and Si is part of the nanomaterial matrix, the frozen tissues were digested and analyzed for content of these two elements. Elemental analysis was performed on an Optima 8300 instrument (PerkinElmer, Waltham, MA). Plasma samples were diluted in 0.1% HNO_3_ prior to injection.

### Pharmacokinetics and biodistribution

The *in vivo* part of the pharmacokinetics studies was performed by Adlego Biomedical AB. Male SD rats were administered a dose of Spago Pix corresponding to 20 µmol Mn/kg by injection in the tail vein. Blood samples were serially collected into heparin tubes at 5, 20, 60 and 120 minutes, centrifuged, and the resulting plasma was frozen at −20°C until analysis. Mn and Si concentrations as biomarkers for the nanomaterial were analyzed by ICP-AES. A smaller study using NMRI mice with blood sampling at 5 and 15 minutes was performed to verify that there were no major species differences in circulation time. For analysis of the biodistribution, MMTV-PyMT mice were euthanized after MR imaging and tumors and femoral skeletal muscles were excised and weighed. One half of muscle and tumor tissues were fixed in 4% paraformaldehyde for immunohistochemistry and the other half was frozen at −20°C for digestion and elemental analysis. For digestion, tumor- and muscle tissues were placed in Teflon containers. Five ml of concentrated HNO_3_ and 5 ml H_2_O were added to each container after which they were heat-treated in a microwave oven at 185°C for 30 minutes. The samples were adjusted to 25 ml with MilliQH_2_O and analyzed for Mn and Si content by ICP-AES.

### MRI

Two separate MR imaging studies were done with a clinical 3 T MRI system (Siemens Magnetom Trio, Siemens Healthcare, Erlangen, Germany) using a clinical wrist coil. In the first study, a fat-saturated turbo spin-echo sequence was used with an echo train length of 3, a repetition time of 350 ms, an echo time of 9.4 ms, and a scan time of 1 min 31 sec. Two-mm slices were imaged with 2 averages and 2.2 mm spacing in a coronal orientation. The receiver bandwidth was 300 Hz/pixel. The acquisition matrix was 256×204 (read × phase) with a field of view of 80×63 mm^2^. In the second study, the spatial resolution and signal-to-noise were increased (acquisition matrix 256×256, field of view of 65×65 mm^2^, 11 averages), and the repetition time was slightly reduced to 320 ms. The scan time was 5 min 30 sec. Anesthesia was given prior to imaging as an intraperitoneal injection of 10 ml/kg of 12.5 mg/ml Ketalar (Pfizer, New York, NY) and 4 mg/ml Rompun (Bayer Animal Health, Leverkusen, Germany). Anesthetized animals were immobilized in a modified 50 ml syringe on top of a 37°C heating pad and placed supine in the wrist coil. After a pre-injection scan, the animals were administered a single tail vein intravenous injection of 10 or 20 µmol Mn/kg of a formulation of 2 mM Mn Spago Pix. Coronal *T*_1_-weighted MR images, covering the whole animal, were acquired before contrast injection and at 10–270 minutes. In cases where the animals were to be re-scanned more than 30 minutes post-dose, they were kept in cages with free access to water and food between imaging sessions.

### Image analysis

MR images were exported as DICOM files and analyzed with the OsiriX software (32-bit Open Source, http://www.osirix-viewer.com). For quantitative analysis of the coronal imaging planes, one region of interest (ROI) was placed in air in the upper left quadrant outside the animal and defined as background. Depending on the availability of well-defined muscle tissue, one or two ROIs were placed separately on either fore- or hind limb muscle tissue. One ROI was placed on each tumor present in the determined image plane. The standard deviation, σ, of the background signal and the mean signal intensity of the muscle and tumor ROIs were determined by the software. The signal-to-noise ratio (SNR) was calculated by dividing the mean signal intensity by σ, and the tumor versus muscle contrast-to-noise ratio (CNR) was calculated by subtracting SNR_muscle_ from SNR_tumor_. Images for visual presentation were treated with global automatic levels settings with GIMP (GNU Image Manipulation Program, version 2.8.2).

### Immunohistochemistry

Paraformaldehyde fixed muscle and tumor tissues collected before injection and at 30 minutes and 2–4 h post-dose were paraffin embedded and thereafter cut (4 µm) and positioned on glass tissue slides. Prior to staining, the slides were placed in an antigen-retrieval solution using an automated pre-treatment module (PT-Link; Dako, Glostrup, Denmark). Slides were stained for PEG in an automated immunohistochemistry robot (Autostainer; Dako) using a primary rabbit anti-PEG-B-47 antibody (Abcam, Cambride, MA) at a concentration of 0.6 µg/ml and a secondary goat-anti rabbit antibody (Dako) at 1∶2000 dilution. Slides were counterstained with hematoxylin, dehydrated and mounted. The stained slides were digitized and total PEG-staining was quantified with the Visomorph software (Visiopharm, Hoersholm, Denmark). Findings in staining patterns were confirmed by a trained pathologist.

### Statistical analysis

Differences between indicated groups were analyzed with unpaired t-tests with the statistical package R (version 3.0.2). For MR CNR quantification, all tumors were treated separately and pooled data from the two studies was analyzed. A p-value of <0.05 was considered statistically significant.

## Results

### Spago Pix nanomaterial

The Spago Pix nanomaterial consists of an organophosphosilane hydrogel with strongly chelated manganese (II) ions and a covalently attached PEG surface layer. It has a globular shape with an average hydrodynamic diameter of 5 nm, as determined both by GPC and DLS measurements. The zeta potential is essentially neutral. The longitudinal relaxivity (r_1_) is 30 mM Mn ^−1^ s^−1^ (60 MHz) in both water and human blood plasma. The transverse relaxivity (r_2_) in human blood plasma is 55 mM Mn ^−1^ s^−1^ (60 MHz). The solutions of Spago Pix used in the *in vivo* experiments had a manganese concentration of 2 mM and a silicon concentration of 62 mM.

### Pharmacokinetics

**Figure 1:**
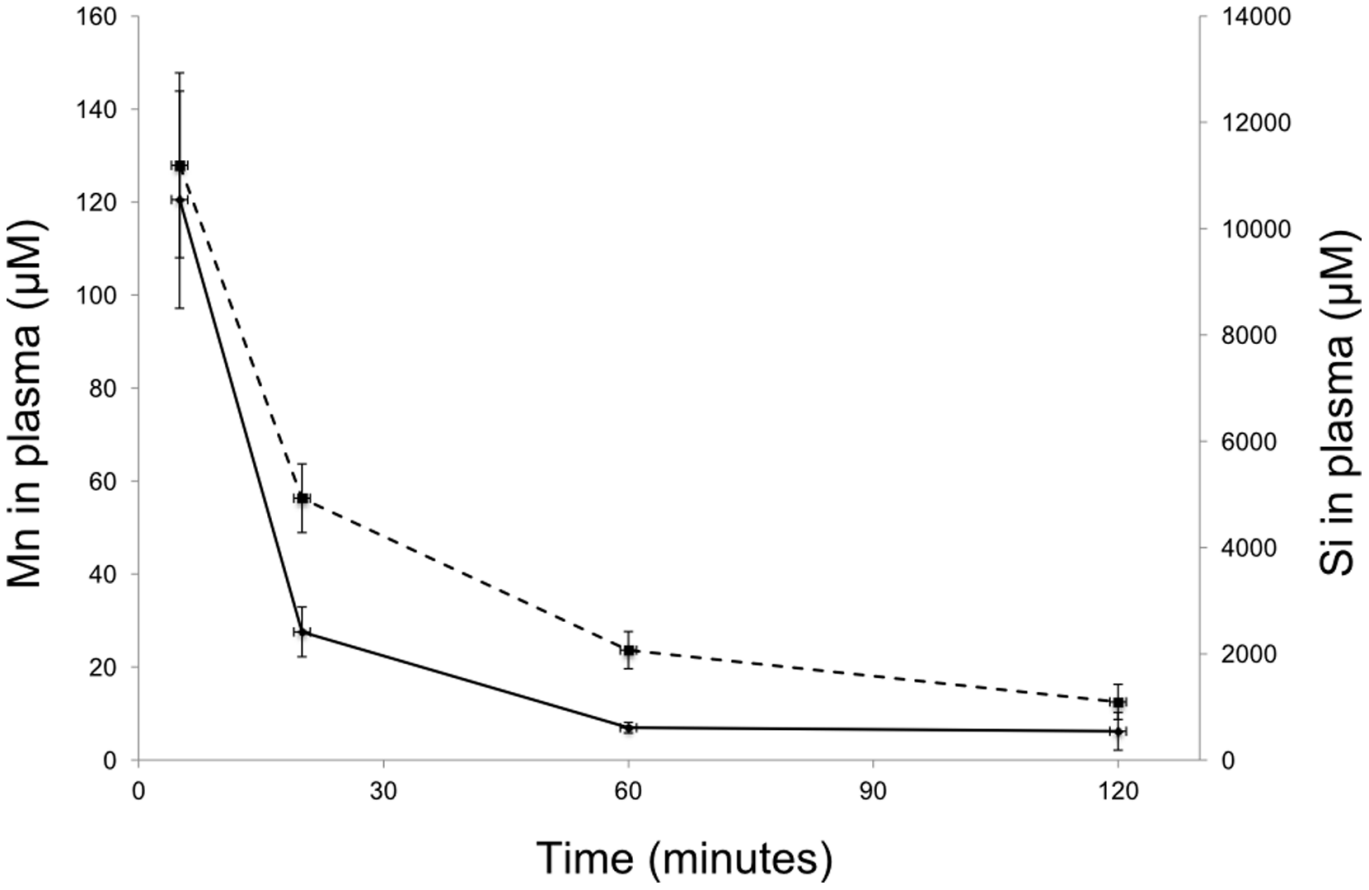
Plasma concentrations of Mn (solid line) and Si (dotted line) in plasma of SD rats injected with Spago Pix (n = 3).

Over the first 60 minutes after injection of Spago Pix, the plasma concentration of both Mn and Si declined by about 90%, with Mn dropping slightly faster than Si. From 60 to 120 minutes both Mn and Si concentrations continued to fall but at a lower rate (Figure 1). Results from a smaller study in mice showed a similar concentration profile (data not shown). Thus, Spago Pix nanoparticles are present in the circulation for more than one hour, defining the time frame in which extravasation to the tumor can occur.

### MRI and CNR quantification

**Figure 2:**
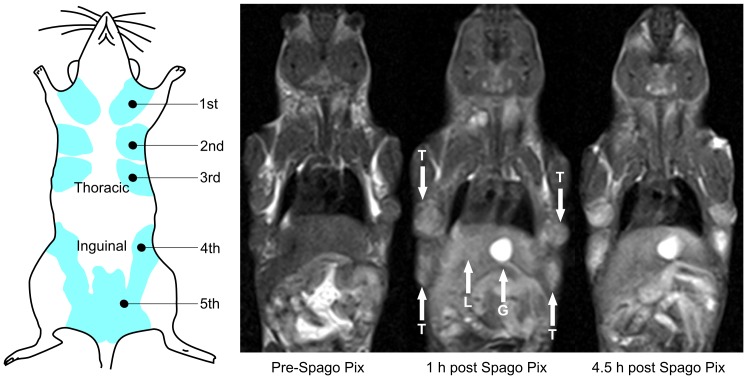
Illustration of mammary gland localization in the mouse and *T*_1_-weighted MR images of pre-injection and 1 h and 4.5 h post injection, respectively, of Spago Pix. The 1 h image has tumors (T), liver (L) and gall bladder (G) indicated with white arrows. Note that the pre-injection image is without applied fat saturation.

**Figure 3:**
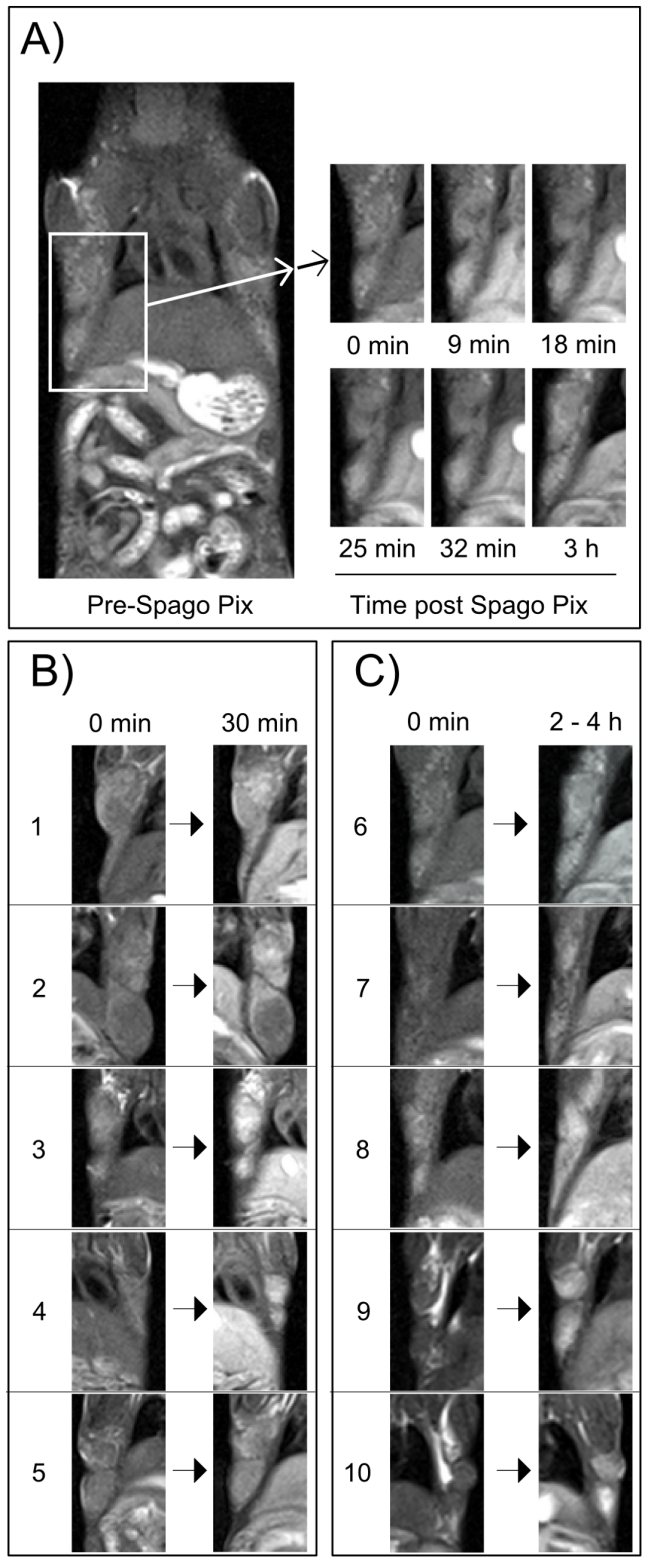
*T*_1_-weighted MR images of the torso of a mouse where enlargements of the indicated tumor site are shown in Panel A before administration of Spago Pix and at five time points thereafter. Panels B and C show enlargements of ten tumor sites (labeled 1–10) in different animals before administration of Spago Pix and 30 min (B) and 2–4 h (C) thereafter.

**Figure 4:**
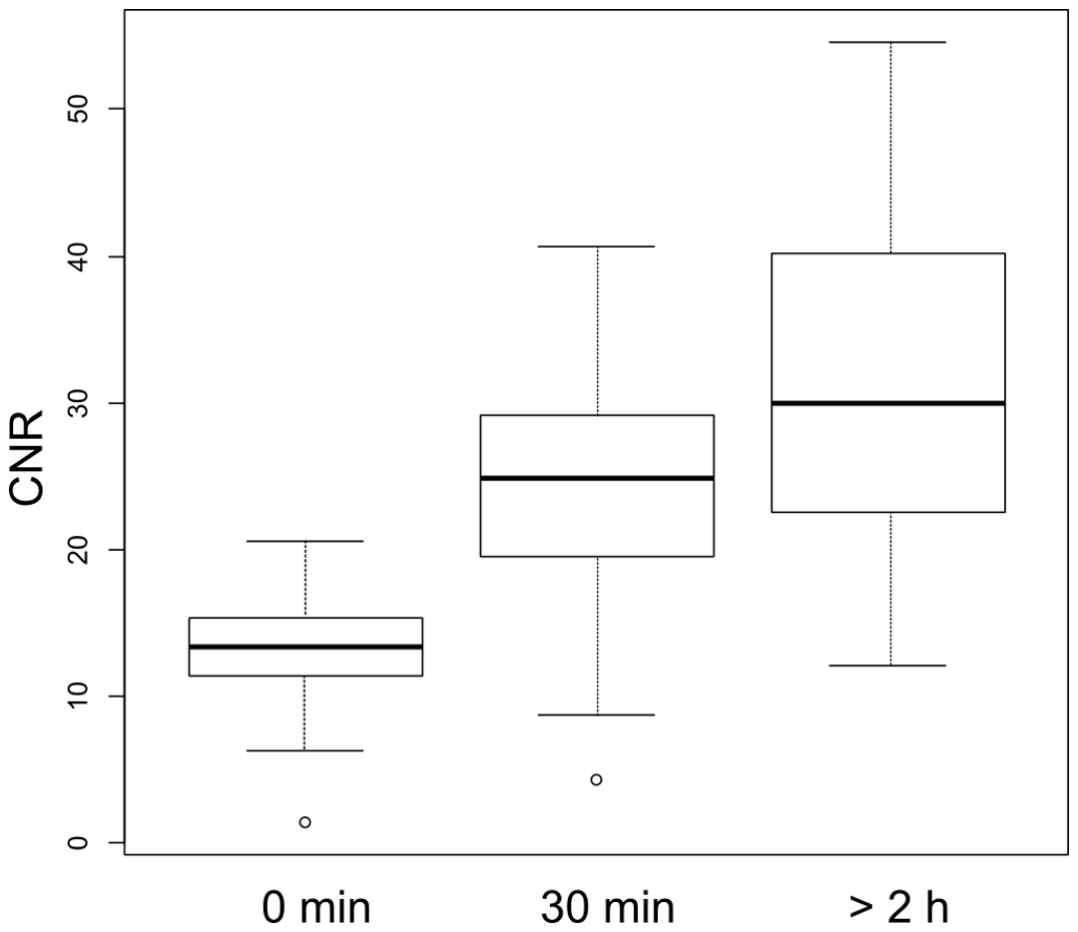
Boxplot of CNR values for pooled tumors at T  = 0 min (24 tumors in 7 animals), 30 min (29 tumors in 7 animals) and >2 h (31 tumors in 6 animals). P_0–30 min_ <0.0001, P_0 min–2 h_ <0.0001, P_30 min–2 h_  = 0.0074.

Between one and nine mammary tissue tumors were detected in all animals included in the two studies. In PyMT animals of the age used in the study (10–12 weeks) tumors occur mostly in the second and third mammary glands (Figure 2). The time frame for when the tumor contrast develops in animals injected with Spago Pix is shown in Figure 3. The two MR imaging studies were performed with slightly different imaging parameters, but the mean CNR of tumors prior to or 30 min and 4.5 h after injection of Spago Pix did not differ significantly between the studies (data not shown). Therefore, CNR data from both studies could be pooled for further analysis. The mean CNR of the tumors prior to administration of Spago Pix was 13.4 (±4.5, n = 24 tumors in 7 animals). Tumor contrast increased within 10 minutes after administration of Spago Pix and the mean CNR 30 min post administration of Spago Pix was 24.4 (±7.9, n = 29 tumors in 7 animals). This corresponds to an 82% increase in CNR. After 2–4.5 h, the mean CNR had further increased to 31.4 (±11.2, n = 31 tumors in 6 animals) corresponding to a 134% CNR increase relative to the pre-dose imaging. A boxplot of CNR values is shown in Figure 4. In addition to an increased tumor CNR, the liver and gall bladder also exhibited increased MR signal intensities.

### Bioanalysis of tumor and muscle tissues

**Figure 5:**
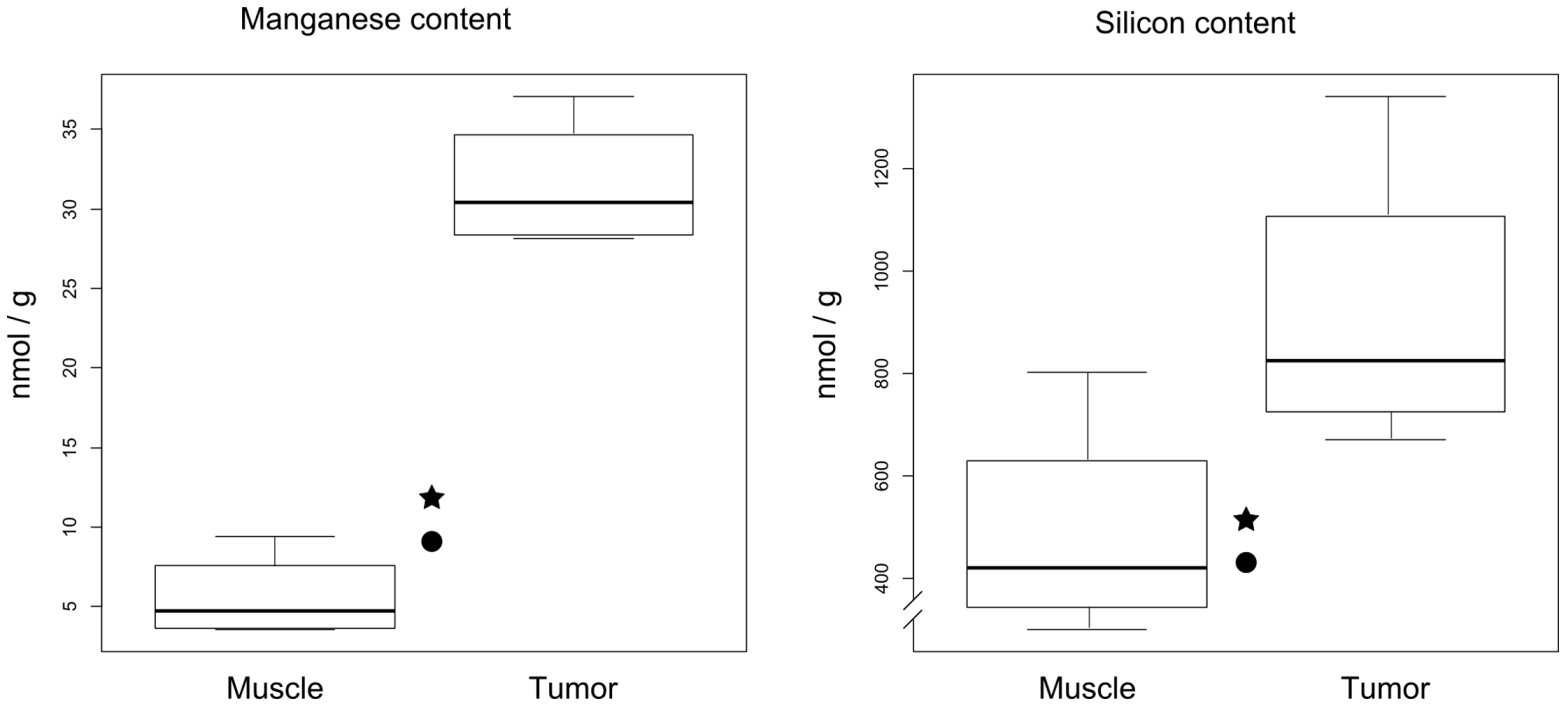
Boxplots of manganese and silicon content in muscle and tumor tissue ∼40 min to 4.5 h post injection of Spago Pix in MRI study 1 (n = 3 for ∼40 min, n = 1 for 4 h). p<0.001 for manganese and p>0.05 for silicon. Background concentrations (n = 1) are indicated by stars (tumor) and circles (muscle). Note the interrupted y-axis in the boxplot for silicon content.

Tumors from MMTV-PyMT mice contained approximately 30 nmol Mn and 800 nmol Si per g tissue, which was 6- and 2-fold higher than in muscle tissue, respectively (Figure 5). Muscle tissues had approximately the same Mn and Si levels as tissues from non-injected animals.

### Immunohistochemistry

**Figure 6:**
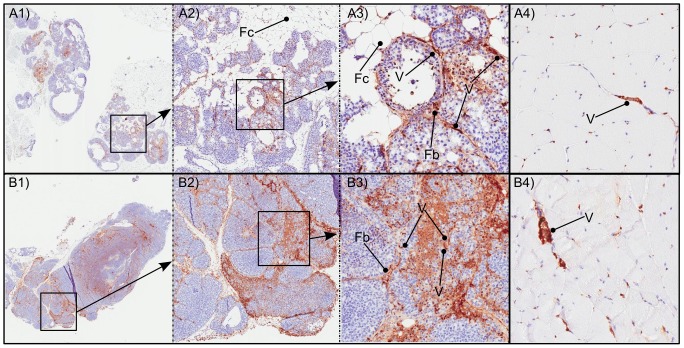
Immunohistochemistry images of anti-PEG (brown) and hematoxylin counterstaining (blue) of tumor (A1–A3 and B1–B3) and muscle tissue (A4 and B4) 30 minutes (A) and 2 h (B) post injection of Spago Pix. Tumor tissue at both time points is shown at three different enlargements where the enlarged areas are indicated by the square boxes. Vessels (V), fibroblasts (Fb) and fat cells (Fc) are indicated with rounded lines. Note that the tumor tissue in panel B1–B3 originates from a mouse injected with a dose of 10 µmol Mn/kg, whereas the other tissues originate from animals administered 20 µmol Mn/kg.

**Figure 7:**
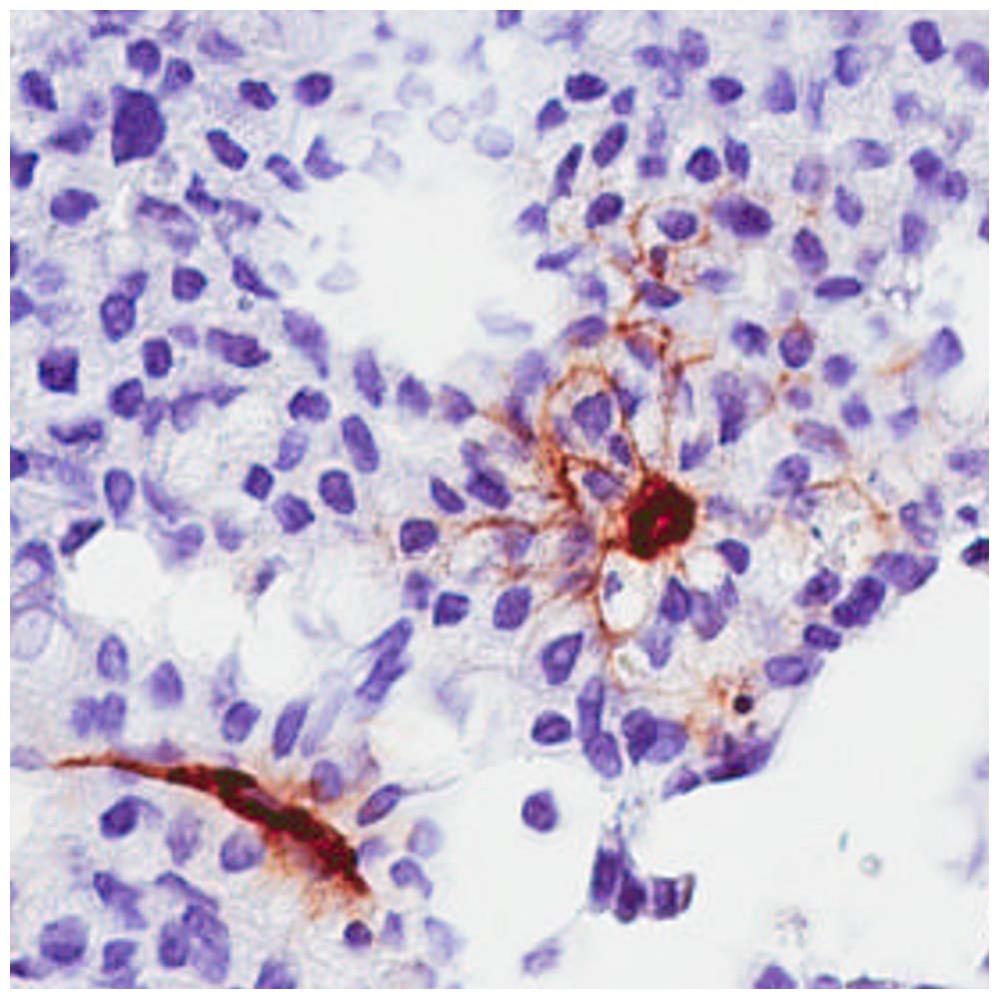
Immunohistochemistry image of anti-PEG (brown) and hematoxylin counterstaining (blue) of tumor tissue 30 min post injection of Spago Pix. The dense PEG-staining corresponds to vessels. Note the staining pattern radiating from the vessel to the right in the figure (see text for details).

**Figure 8:**
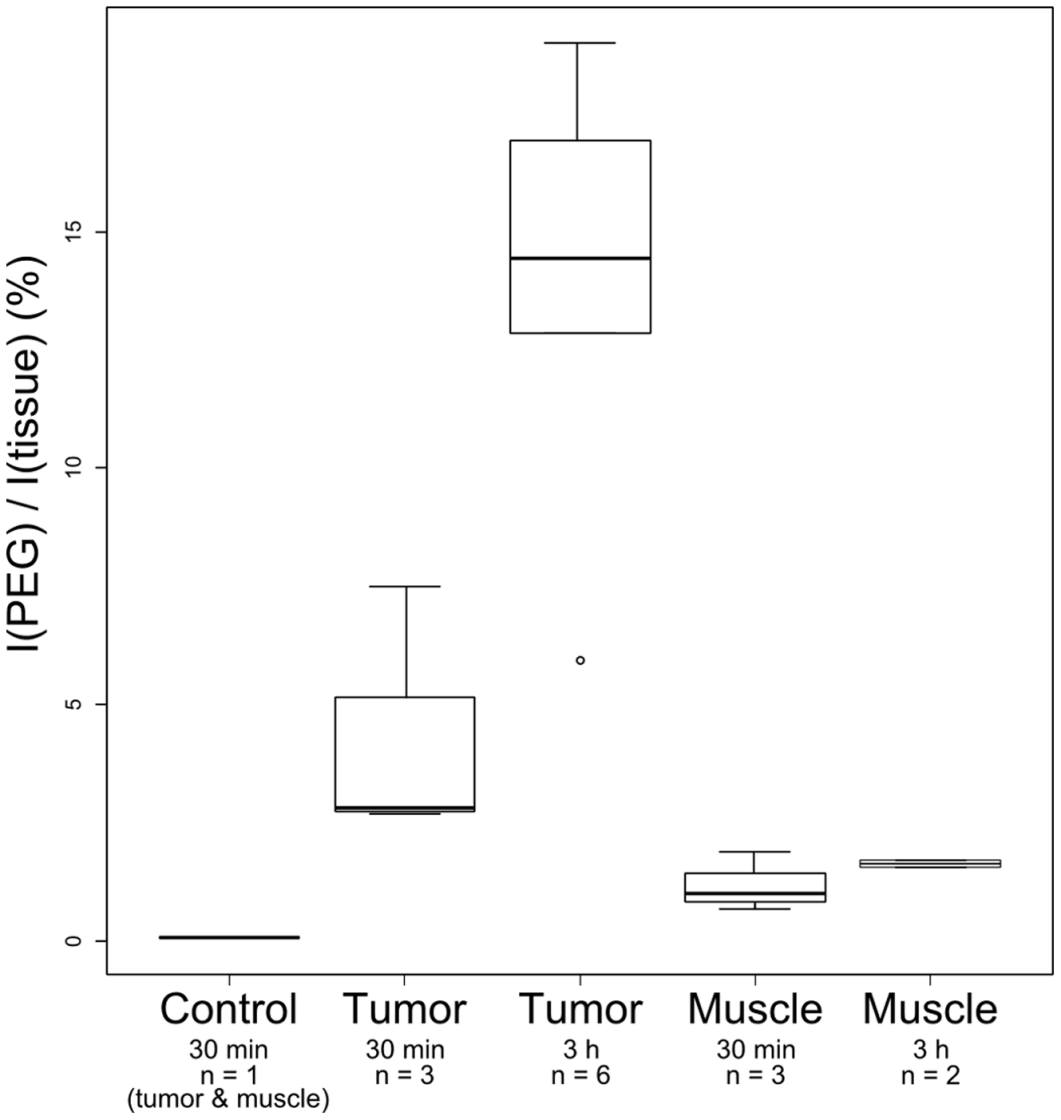
PEG surface area in tumor and muscle at 30 minutes and 2–4 hours post-injection of Spago Pix. p<0.01 for tumor at 2–4 h compared to muscle at 30 min and 2–4 h and compared to tumor at 30 min. The difference between tumor at 30 min and the muscle groups was not statistically significant.

The area of PEG (a coating constituent of Spago Pix) staining was large in tumoral areas, especially in stromal tissues between tumor foci. Specifically in these areas, vessels and fibroblasts where strongly stained. Several tumor foci also showed PEG-positive signals, and the area of those foci was larger at the later time points. In contrast, staining in muscle tissue was limited to the area inside vessels between muscle structures (Figure 6). Tumor vessels frequently showed signs of extravasation of Spago Pix into surrounding tumor tissue (Figure 7) in contrast to vessels in muscle tissue in which Spago Pix was contained within the vessels (Figure 6, panels A4 and B4). The PEG staining area was larger at the later time points (2–4 h) compared to 30 minutes after injection as measured by image analysis (Figure 8). In contrast, muscles showed only a weak increase in PEG staining. The appearance of the staining in tumors was more focal at the early time point and more widespread throughout the tumor at the later time points.

## Discussion

By combining a macromolecular structure with the relatively safer Mn as a contrast enhancer, our ambition was to create a CA with broad tumor selectivity and a very good safety profile. It should also allow for determination of vascular leakage and tumor grade. Indeed, using the prototypic macromolecular CA albumin-(Gd-DTPA), others have demonstrated that contrast enhancement correlated with histologic tumor grade in preclinical models [13]. In a clinical setting, ultra-small paramagnetic iron oxide (USPIO) particles (NC100150/Clariscan, Nycomed) were selectively taken up by malignant but not benign breast tissue lesions [13], [14]. Such iron oxide containing USPIOs (e.g. Clariscan, Sinerem and Supravist) normally give negative contrast, and their clinical utility have been limited to blood pool agents for magnetic resonance angiography where positive contrast can be enforced by extremely *T*_1_-weighted imaging sequences [15]. To obtain more efficient blood pool agents with positive contrast, Gd-containing macromolecular CAs, including dendrimers and various copolymers [8] have been generated and shown promising results in preclinical models (e.g. Gadomer-17 [16]). Another approach to create macromolecular blood pool agents with positive contrast was to modify small molecule Gd-chelators to bind albumin, e.g. MS-325 (gadofosveset trisodium, Ablavar) and thereby form a macromolecular complex. Intratumoral levels of MS-325 correlated with histopathology in a rat model [17]. However, the clinical utility of MS-325 has been questioned due to a relatively complex kinetic behavior and the finding that the agent is taken up in healthy lymph nodes rather than in tumor containing micrometastases [18]. MS-325 is currently on the market and used for aortoiliac occlusive disease, and it is also in clinical trials for e.g. staging of lymph nodes (www.clinicaltrials.gov).

The Spago Pix nanomaterial is neutral in charge and has an average hydrodynamic diameter of 5 nm. This size was chosen as a compromise between tumor selectivity and renal filtration, as it should give approximately 50% renal filtration [19]. Mn as contrast enhancer has a more attractive safety profile than e.g. Gd, especially when complexed [20], [21]. Prolonged environmental exposure to high doses of Mn, for instance shown for manganese mine workers, can cause neurotoxicity, but this is generally not considered an issue with intravenous injections of Mn-containing CAs [22]. The longitudinal relaxivity (r_1_) of Spago Pix is 30 mM Mn ^−1^ s^−1^ (60 MHz) in both water and human blood plasma, which is significantly higher than the clinically used contrast agents [23].

MR analysis of tumor-bearing MMTV-PyMT mice injected with Spago Pix showed that tumor to muscle contrast developed over a couple of hours. This corresponds to a first phase where the nanomaterial enters the tumor and a second phase where the background is washed out leading to a gradual increase in CNR. The MRI images in figures 2 and 3 show enhanced signal in the liver and gall bladder, which indicates the presence of Spago Pix in these areas. A comprehensive distribution and excretion study is currently being prepared, but preliminary unpublished data indicate that manganese is separated from the nanostructure in the liver and is excreted via the bile and feces whereas the nanostructure matrix is excreted mainly by renal filtration. This is also the reason for the differences between the plasma concentrations of manganese and silicon seen in figure 1. Thus, our current interpretation is that the contrast enhancement in the liver and gall bladder is partly due to the presence of manganese which has been extracted from Spago Pix. The 82% increase of tumor contrast after 30 minutes and 134% after 2–4 hours resulted in good tumor visualization, and importantly, all animals showed enhancement. The implication of this is that also very small tumors should be detectable with high sensitivity.

The MMTV-PyMT model closely mimics human breast cancer at the histological and molecular levels [24]. The PyMT oncogene drives the development of well-differentiated, luminal-type adenomas that progress to metastatic, poorly-differentiated adenocarcinomas [25]. The PyMT-model would therefore be suitable for the analysis of contrast accumulation in benign versus malignant lesions, but as benign adenomas in this model are primarily found at an early age (4–8 weeks), and the animals studied here were 10–12 weeks old, the hypothesis of selective leakage into malignant lesions was not addressed.

Immunohistochemical analysis of PEG staining in tissues showed that over time, the nanomaterial accumulated significantly more in tumor tissue than in muscle tissue, closely matching the tumor MR contrast development. Interestingly, at the early time point the PEG staining was often localized in stromal tissue near blood vessels, whereas after several hours it was more spread out into tumor foci, indicating that Spago Pix penetrates deeper into the tumor tissue over time. Only minor PEG staining was detected in muscle tissues demonstrating lack of vascular leakiness in this tissue. Analysis of Mn and Si further corroborated the specific accumulation of nanomaterial in the tumors. Taken together, MRI, IHC, and elemental analysis strongly support that Spago Pix selectively accumulates in tumors via the EPR effect and thereby facilitates tumor detection on the MR images.

Reports from preclinical studies have shown that the accumulation of macromolecular CAs is affected by drugs directed against tumor vascularization [26], [27]. Given the finding that Spago Pix selectively leaks into areas of tumor tissue, most likely as a result of the increased vascular permeability frequently seen in tumors, Spago Pix has potential, not only as a diagnostic and/or prognostic agent, but also in monitoring anti-angiogenic therapy.

In conclusion, MR imaging of tumor-bearing MMTV-PyMT mice injected with novel MRI CA Spago Pix showed a significant increase in contrast enhancement for tumor versus muscle at 30 minutes post-administration which was retained and further increased 2–4 hours after administration. The long retention of Spago Pix in tumor tissue should allow for a more generous imaging time frame in the clinical setting. Based on what has been observed with other macromolecular contrast agents [13], [14], Spago Pix may be expected to allow characterization of lesions as benign or malignant.
